# Submandibular gland involvement in oral cavity squamous cell carcinoma: a retrospective multicenter study

**DOI:** 10.1007/s00405-023-08007-8

**Published:** 2023-06-06

**Authors:** Oreste Iocca, Chiara Copelli, Paolo Garzino-Demo, Guglielmo Ramieri, Stefano Rubattino, Luca Sedran, Fabio Volpe, Alfonso Manfuso, Francesco Longo, Gregorio Sanchez-Aniceto, Álvaro Rivero-Calle, Aitor García-Sánchez, Raul Pellini, Gerardo Petruzzi, Silvia Moretto, Laith Al-Qamachi, Hiba Aga, Stephen Ridley, Pasquale Di Maio

**Affiliations:** 1 Division of Maxillofacial Surgery, Città della Salute e della Scienza Hospital, University of Torino, Turin, Italy; 2 Operative Unit of Maxillofacial Surgery, Otolaryngology and Dentistry, Fondazione IRCCS Casa Sollievo della Sofferenza, San Giovanni Rotondo, FG Italy; 3 12 de Octubre University Hospital, Av. de Cordoba S/N, 28041 Madrid, Spain; 4 Department of Maxillofacial Surgery, Hospital Quiron Salud, Ciudad Real, Spain; 5 Department of Otolaryngology - Head and Neck Surgery, IRCCS Regina Elena National Cancer Institute, Rome, Italy; 6 Queen’s Medical University Hospital, Nottingham, UK; 7 Department of Otolaryngology - Head Neck Surgery, Hospital of Magenta, Milan, Italy; 8 Division of Maxillofacial Surgery, Department of Interdisciplinary Medicine, University of Bari, Bari, Italy

**Keywords:** Oral cancer, Submandibular gland, Head and neck cancer, Oral squamous cell carcinoma, Neck dissection, Submandibular gland invasion, Meta-analysis, Systematic review

## Abstract

**Background:**

The submandibular gland (SMG) is routinely excised during neck dissection. Given the importance of the SMG in saliva production, it is important to understand its involvement rate by cancer tissue and the feasibility of its preservation.

**Methods:**

Retrospective data were collected from five academic centers in Europe. The study involved adult patients affected by primary oral cavity carcinoma (OCC) undergoing tumor excision and neck dissection. The main outcome analyzed was the SMG involvement rate. A systematic review and a meta-analysis were also conducted to provide an updated synthesis of the topic.

**Results:**

A total of 642 patients were enrolled. The SMG involvement rate was 12/642 (1.9%; 95% CI 1.0–3.2) when considered per patient, and 12/852 (1.4%; 95% CI 0.6–2.1) when considered per gland. All the glands involved were ipsilateral to the tumor. Statistical analysis showed that predictive factors for gland invasion were: advanced pT status, advanced nodal involvement, presence of extracapsular spread and perivascular invasion. The involvement of level I lymph nodes was associated with gland invasion in 9 out of 12 cases. pN0 cases were correlated with a reduced risk of SMG involvement. The review of the literature and the meta-analysis confirmed the rare involvement of the SMG: on the 4458 patients and 5037 glands analyzed, the involvement rate was 1.8% (99% CI 1.1–2.7) and 1.6% (99% CI 1.0–2.4), respectively.

**Conclusions:**

The incidence of SMG involvement in primary OCC is rare. Therefore, exploring gland preservation as an option in selected cases would be reasonable. Future prospective studies are needed to investigate the oncological safety and the real impact on quality of life of SMG preservation.

**Supplementary Information:**

The online version contains supplementary material available at 10.1007/s00405-023-08007-8.

## Introduction

Surgical dissection of the neck is a mainstay of management in patients affected by oral cavity carcinoma (OCC), as nodal metastases are considered the most important prognostic factor [1]. Neck dissection techniques have evolved considerably since the first description of radical dissection by Crile in 1906 [2]. Nowadays, modified radical and selective neck dissections are the standard of care for most patients. The decision to perform a therapeutic neck dissection is straightforward in node-positive patients, on the other hand recent evidence points to greater survival benefits and locoregional control (LRC) when elective neck dissection is performed in N0 cases [3]. The most common nodal levels involved in oral cavity cancer are level I, II, and III, although more advanced disease can involve levels IV and V as well [4, 5]. When level I is included in the dissection, as is the case in most oral cavity cancers, the submandibular gland (SMG) is routinely excised. However, the current available evidence seems to show a rare rate of SMG involvement in OCC and thus some authors have advocated for the preservation of the gland [6]. The SMGs are an important component of the healthy physiology of the oral cavity, because they produce most of the unstimulated saliva over 24 h [7]. Preservation of one or both SMGs can potentially reduce the occurrence of xerostomia, which is one of the OCC treatments sequelae that greatly impairs patients’-related quality of life. To identify the rate of SMG involvement, the pattern of invasion, and the pathological characteristics of involvement, the authors decided to conduct a multicenter, retrospective study from five academic centers in Europe. The aim of this study was to collect data from the largest sample of patients published so far and identify the rate of SMG involvement. The results observed were compared with an updated quantitative synthesis of the literature published until completion of this study, obtained through a systematic review and meta-analysis.

## Materials and methods

### Retrospective study

The present study involved adult patients with a primary OCC diagnosis that underwent both tumor excision and neck dissection in the same operation between 2017 and 2021. Exclusion criteria were: previously treated head and neck malignancy, previous irradiation in the area, delayed neck dissection. Five academic tertiary care centers in Europe were included in the study: University Hospital of Torino, Hospital Casa Sollievo della Sofferenza, 12 de Octubre Hospital, Regina Elena National Cancer Institute, Queen’s Medical Hospital of Birmingham. The data collection started from the histopathological reports of OCC which reported the presence of the submandibular gland in the examined sample, and then integrated them with the data of the surgical reports. The data obtained were collected on a database common to all the centers involved. Care was taken to preserve the identity and sensitive data of the included patients.

A statistical analysis has been performed to evaluate if cases of SMG involvement vary significantly according to primary sub-site, pN, pT, extracapsular spread (ECS), perivascular invasion (PVI), perineural invasion (PNI), and neck levels involved. All tests were two-sided. Chi-square tests were performed for categorical variables and analysis of adjusted residuals was performed to better interpret statistically significant results. Statistical significance was set as *p* < 0.05. Analyses were performed with SPSS 21.0 (SPSS inc.).

### Systematic review and meta-analysis

A systematic review and a meta-analysis were conducted according to the PRISMA checklist [8]. A literature search on PubMed, Embase and Scopus, was performed up to January 1st, 2022. No language restriction was applied. The literature search and subsequent analysis was focused on papers reporting submandibular gland involvement in OCC. We excluded case reports and studies including less than 10 patients. Data extraction was performed by two investigators (OI and PDM), who searched for studies independently. Identification of studies was performed through screening of the titles and selecting the abstracts for full-text inclusion. The reviewers screened all the abstracts and their suitability for the subsequent analysis according to the pre-specified inclusion and exclusion criteria. Single arm meta-analysis of SMG involvement rates was conducted “per patient” and “per gland excised”. Meta-analyses were done using the R software for statistical computing (R 2.10.1; “meta” package). Arcsine transformation of the data was performed for the analysis on overall detection rates, a 99% confidence interval (CI) was chosen for calculations. Restricted maximum likelihood was the method used for the random effects meta-analysis on overall SMG involvement rate. The modified Newcastle–Ottawa Scale (mNOS) was applied by two authors (O.I. and P.D.M.) to estimate the risk of bias in the included studies.

## Results

### Retrospective study

A total of 642 patients were included (Table 1), 356 males and 286 females. Mean age was 64.7 years (95% CI 63.7–65.7). Since also contralateral neck dissections were considered, a total of 852 glands were analyzed. The most common tumor site was the tongue (43.9% of the cases), followed by mandibular alveolar ridge (17.6%), floor of mouth (14.3%), buccal mucosa (10.3%), retromolar trigone (8.1%), maxillary alveolar ridge (3.9%), and hard palate (1.9%). Contralateral neck dissection was performed on 210 patients. En bloc resection of the primary tumor and cervical lymph nodes was performed in 192 cases. Most of the patients underwent resection with microvascular reconstruction (56.4%). A total of 12 glands were involved by cancer. For this reason, when considered per patient, the involvement rate was 12/642 (1.9%; 95% CI 1.0–3.2). When considered per gland, the involvement rate was 12/852 (1.4%; 95% CI 0.6–2.1%). All the cases were ipsilateral to the side of the tumor (Table 2). The majority of SMG had direct invasion as the type of involvement, followed by extracapsular spread from level Ib lymph node, two cases showed the invasion from intraglandular lymph nodes (Table 3). Ten out of twelve patients with SMG involved were pN + , the remaining two cases were pN0 in which large tumors directly invaded the submandibular gland. Ten out of twelve patients with SGM involvement were classified as pT3–4.

**Table 1:** Demographical and pathological characteristics of the included patients

Variable	Frequency (relative percent)	Mean (95% confidence interval)
Age		64.7 (63.7–65.7)
Gender		
Male	356	
Female	286	
Primary sub-site		
Tongue	282 (43.9%)	
Mandibular alveolar ridge	113 (17.6%)	
Floor of mouth	92 (14.3%)	
Buccal mucosa	66 (10.3%)	
Retromolar trigone	52 (8.1%)	
Maxillary alveolar ridge	25 (3.9%)	
Hard palate	12 (1.9%)	
pN		
pN0	341 (53.1%)	
pN1	70 (10.9%)	
pN2a	24 (3.7%)	
pN2b	90 (14%)	
pN2c	16 (2.5)	
pN3b	101 (15.7%)	
pT		
pT1	67 (10.4%)	
pT2	209 (32.6%)	
pT3	182 (28.3%)	
pT4a	179 (27.9%)	
pT4b	5 (0.8%)	
Pathological depth of invasion		10.9 mm (10.3–11.4)
Neck dissection		
Omolateral	642	
I–III	133 (20.7%)	
I–IV	299 (46.6%)	
I–V	210 (32.7%)	
Contralateral	210	
I–III	84 (40%)	
I–IV	93 (44.3%)	
I–V	33 (15.7%)	
Surgery performed		
Resection with microvascular flap reconstruction	362 (56.4%)	
Resection with local flap reconstruction	154 (24.0%)	
Resection	126 (19.6%)	
Extracapsular spread		
No	422 (65.7%)	
Yes	217 (33.8%)	
NA	3 (0.5%)	
Perivascular invasion		
No	490 (76.3%)	
Yes	149 (23.2%)	
NA	3 (0.5%)	
Perineural invasion		
No	370 (57.6%)	
Yes	268 (41.7%)	
NA	4 (0.6%)	
Submandibular gland involvement		
No	630 (98.1%)	
Yes	12 (1.9%; 95% CI 1.0–3.2)

**Table 2:** Statistical analysis of the predictive factors of submandibular gland involvement

Variable	Frequency	Submandibular gland not involved	Submandibular gland involved	*p* value
Patients	642	630	12	
Primary sub-site				
Tongue	282	281	1^§^	
Mandibular alveolar ridge	113	111	2^§^	
Floor of mouth	92	86	6^§^	
Buccal mucosa	66	65	1	
Retromolar trigone	52	50	2	
Maxillary alveolar ridge	25	25	0	
Hard palate	12	12	0	0.012*
pN				
pN0	341	339^§^	2	
pN1	70	68	2	
pN2a	24	23	1	
pN2b	90	89	1	
pN2c	16	15	1	
pN3b	101	96	5^§^	0.046*
pT				
pT1	67	67	0	
pT2	209	207	2	
pT3	182	180	2	
pT4a	179	172	7^§^	
pT4b	5	4	1^§^	0.013*
Extracapsular spread				
No	422	418^§^	4	
Yes	217	210	7^§^	
NA	3			0.036*
Perivascular invasion				
No	490	485^§^	5	
Yes	149	143	6^§^	
NA	3	2	1	0.024*
Perineural invasion				
No	370	365	5	
Yes	268	263	5	
NA	4			0.534
Levels involved in pN + patients				
I	65	60	5	
II	48	48	0	
III	26	26	0	
IV	15	15	0	
I–II	32	31	1	
I–III	16	15	1	
II–III	20	20	0	
I and III	5	5	0	
I and IV	4	4	0	
I–IV	9	7	2	
I–V	3	3	0	
II–IV	13	13	0	
II–V	1	1	0	0.11

Bivariate analysis performed through *χ*^2^ test. Interpretation of statistical results performed through the analysis of adjusted residuals

*p value less than 0.05 meaning a statistically significant result

^§^Adjusted residual showing the statistically significant variable

**Table 3:** Characteristics of the cases in which there was a SMG involvement

Age	Sex	Anatomic location	pN	pT	DOI	ECS	PNI	PVI	Type of surgery	Type of SMG involvement
47	Male	Floor of mouth	pN3 b	pT2	8	Yes	No	No	Resection	Extracapsular spread from level IB lymph node
68	Female	Tongue	pN3 b	pT4 a	20	Yes	Yes	No	Resection with microvascular flap reconstruction	Direct invasion
65	Male	Floor of mouth	pN0	pT4 a	NA	No	No	No	Resection with microvascular flap reconstruction	Direct invasion
66	Female	Retromolar trigone	pN2 a	pT4 a	NA	No	Yes	No	Resection with microvascular flap reconstruction	Intraglandular lymph node
43	Male	Floor of mouth	pN3 b	pT2	7	Yes	No	Yes	Resection with local flap reconstruction	Direct invasion
78	Male	Floor of mouth	pN1	pT4 b	NA	NA	NA	NA	Resection with microvascular flap reconstruction	Direct invasion
73	Female	Buccal mucosa	pN1	pT3	NA	Yes	Yes	Yes	Resection with microvascular flap reconstruction	Direct invasion
69	Female	Mandibular alveolar ridge	pN3 b	pT4 a	10	Yes	No	Yes	Resection with microvascular flap reconstruction	Extracapsular spread from level IB lymph node
64	Male	Floor of mouth	pN3 b	pT3	23	Yes	Yes	Yes	Resection with microvascular flap reconstruction	Extracapsular spread from level IB lymph node
57	Male	Retromolar trigone	pN2 b	pT4 a	NA	Yes	Yes	Na	Resection with microvascular flap reconstruction	Intraglandular lymph node
42	Male	Floor of mouth	pN2 c	pT4 a	18	No	Yes	Yes	Resection with microvascular flap reconstruction	Direct invasion
65	Male	Mandibular alveolar ridge	pN0	pT4 a	15	No	No	No	Resection with microvascular flap reconstruction	Direct invasion

DOI, depth of invasion; ECS, extracapsular spread; PNI, perineural invasion; PVI, perivascular invasion

Statistical analysis of the predictive factors of submandibular gland involvement showed that cancer of floor of the mouth is significantly associated with SMG involvement (*p* < 0.05, adjusted residual 3.6). Advanced pT status, specifically T4a and T4b were associated with the SMG involvement (*p* < 0.05, adjusted residual 2.4 and 3.0, respectively). In addition, advanced nodal involvement status was significant, specifically pN3b (*p* < 0.05, adjusted residual 2.5). The same was found for ECS involvement (*p* < 0.05, adjusted residual 2.1), and for PVI (*p* < 0.05, adjusted residual 2.5). Level I involvement was associated with SMG involvement (adjusted residual 2.1), the same for multiple levels involved (specifically level I–IV *p* value adjusted residual 3.3), although the low number of events did not allow to reach a statistical significance. In addition, pN0 cases were associated with a reduced risk of SMG involvement (*p* < 0.05, adjusted residual 2.6).

### Systematic review and meta-analysis

A total of 24 studies [9–31, 33] were identified from the systematic review of the literature. Most of the studies were retrospective (20/24), while 3 were prospective (3/24). In total, 4458 patients and 5037 glands were analyzed.

**Fig. 1:**
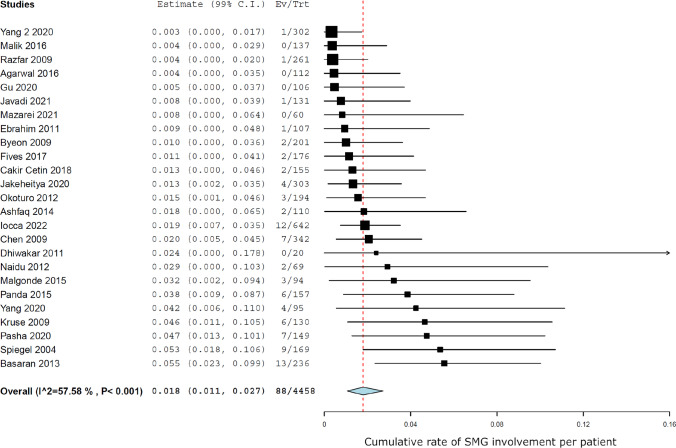
Forest plot showing the cumulative rate of SMG involvement per patient

**Fig. 2:**
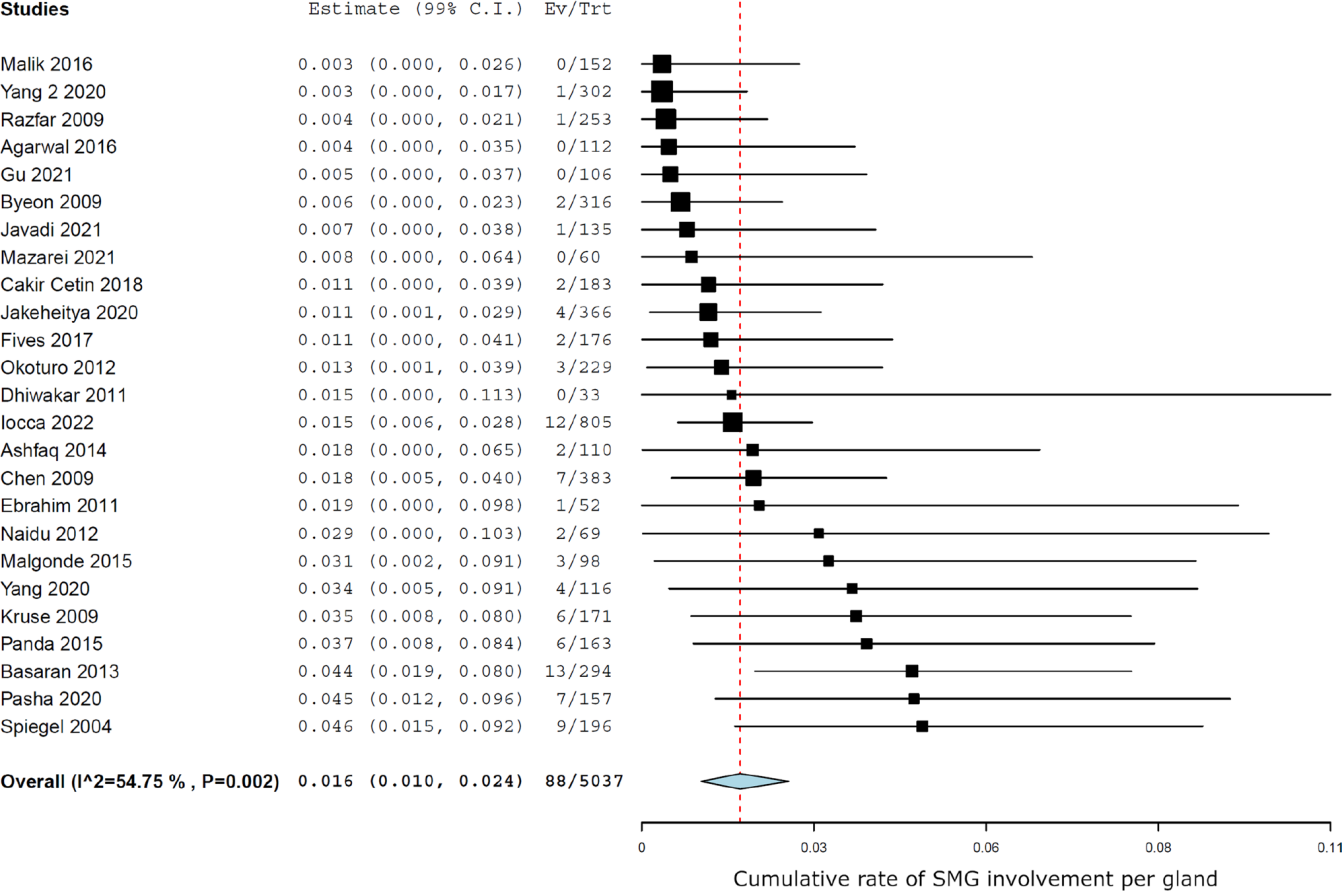
Forest plot showing the cumulative rate of SMG involvement per gland

Meta-analysis showed a rate of involvement of 88 glands out of a total of 4458 patients, which means that the cumulative SMG involvement rate per patient was 1.8% (99% CI 1.1–2.7) (Fig. 1), while SMG involvement was 1.6% (99% CI 1.0–2.4) when considered per gland (Fig. 2). Heterogeneity was moderate (*I*^2^ 57.5%, *p* < 0.005).

Through the application of the mNOS, most of the included studies (*n* = 17, 70%) were considered to be at a low risk of bias. The remaining seven studies were deemed to have a moderate risk of bias. The results of the risk of bias assessment are shown in Supplementary Material Table S1.

## Discussion

The aim of this study was to estimate the involvement of the submandibular gland in patients affected by squamous cell carcinoma of the oral cavity and to explore the possibility of its preservation during neck dissection. Given that the SMG lies in level Ib, the analysis of the oncological safety of leaving the gland in situ could have important clinical implications. Multiple authors [13, 16, 21] advocate for the preservation of the gland when dissection of level I is included in the treatment plan. This relies on the observation that xerostomia is one the most debilitating symptoms for the patients, impairing their quality of life after head and neck cancer treatment [32]. Preservation of one or both submandibular glands could help greatly in reducing the incidence of this complication. Gu et al. [33]. evaluated the impact of submandibular gland preservation in the neck management of early stage OCC, the authors studied 31 patients in which the gland was preserved and compared them to 131 patients in which the submandibular gland was routinely removed. The results showed differences in quality of life in terms of subjective feeling of saliva production, chewing capacity, and swallowing outcomes. Moreover, the saliva flow rate in the preservation group remained significantly higher than in the excision group after 1 year of follow-up. The same authors evaluated the survival outcomes of SMG preservation versus excision, and they did not find any difference in loco-regional recurrence or disease-specific survival between the two groups of patients.

It is likely that gland sparing reduces the incidence of damage to the hypoglossal and lingual nerves and to the marginalis branch of the facial nerve. Furthermore, gland preservation makes the dissection and ligation of the facial vessels unnecessary, keeping intact and preventing damage to vascular structures that can be useful for immediate reconstructive purposes or future interventions.

To conduct a SMG preservation safely, it is, however, important to perform a careful dissection of the whole fibroadipose tissue surrounding the gland and containing the lymph nodes of the level Ib. Dhiwakar et al. [15]. described in detail the technique and surgical steps useful for a safe gland preservation procedure. The Authors carried out the surgery on 30 neck dissections in which level Ib was included; they carefully removed the fibroadipose tissue around all the borders of the gland while preserving the facial artery, the facial vein, and the visible branches of the gland. The gland was removed for examination in a second step. In all the examined procedures, a complete lymph node removal was achieved, with 4 cases presenting foci of metastatic carcinoma and no gland involvement by pathology. The Authors also evaluated the potential damage to the marginalis branch of the facial nerve, finding that in just two cases there was a persistent impairment of the nerve function beyond 6 months.

To the best of our knowledge, our pool of 642 patients and 852 glands analyzed constitute the largest clinical study on this topic so far. The involvement of 12 SMGs, or a rate of 1.9% per patient, confirms that SMG invasion in OCC is a rare occurrence. The statistical analysis shed light on the predictors of SMG involvement. Unsurprisingly, given that direct invasion is the most common way that tumors can spread to the gland, localization of the cancer to the floor of the mouth resulted in a higher risk of gland invasion. Further predictive factors were level I node positivity, which can determine a gland invasion by direct spreading of the tumor from adjacent lymph nodes, and advanced T stage. Interestingly, two cases were characterized by the presence of intraglandular lymph nodes, a rare occurrence which has been already reported in the literature [17]. In the patients who underwent a bilateral neck dissection, no involvement of the SMG was observed contralateral to the side of the tumor.

The outcomes of the study correlate well with the results of the meta-analysis, in which all the available evidence on the topic has been collected and quantitatively synthesized. From there it emerges that the SMG invasion was detected in 88/4458 patients cumulatively, equaling only 1.8% of the cases.

Combining the results of the retrospective data collected and the meta-analysis of the literature, it is reasonable to assume that in the following cases the SMG can be preserved during neck dissection: early stage carcinomas, tumors not arising from the floor of the mouth, no involvement of the level Ib lymph nodes and neck dissection contralateral to the side of the tumor. In addition, it is intuitive that if there is a suspicion of direct extension to the submandibular duct it is likely that the whole gland with its ductal system should be removed for oncological safety.

As regards the Tumor–Node (T–N) tract, recent literature agrees that its surgical dissection is associated with a better prognosis in advanced forms of squamous carcinoma of the tongue and floor of the mouth [34, 35]. In such cases, preservation of the submandibular gland can lead to a non-oncologically safe resection of the T–N tract. In the present study, stages T4a and T4b were, in fact, associated with a greater risk of involvement of SMG (*p* < 0.05). For these reasons, preservation of SMG should be considered unsuitable in advanced T stage (T3–4) cancers. In contrast, no significant differences were observed in disease-free survival of T1–T2 tumors treated with or without T–N block resection [36], so gland preservation strategies could be implemented in these early T stage cases.

The detection that the SMG rarely is affected by tumor invasion can have implications also in cases where neck irradiation is planned. Once the oncological safety is established, a gland sparing irradiation protocol could become a routine procedure in selected cases to preserve the salivary flow [37]. Different Authors have proposed this over the last decade [38, 39]. Recently, Varra et al. [40]. examined the possibility of selectively sparing the SMG in patients affected by T1–T2, N0–N3, oral cavity or oropharynx carcinoma that were treated with upfront or postoperative radiotherapy (RT). They selected 32 SMG to be contoured during the intensity modulated radiotherapy (IMRT) treatment planning. The mean dose to the spared SMG was 58.9 Gray (Gy) versus the 66.6 Gy to the glands that were not spared. The Authors confirmed the feasibility of a gland preservation protocol, finding no differences in prognosis between the two groups. Although the number of patients that developed acute or late xerostomia was lower in the spared group, the difference was not statistically significant.

The strengths of our study are numerous. First, the large sample size enabled us to draw potentially reliable estimates of the real incidence of SMG involvement in OCC. Second, the multicenter design of the study made it possible to study a variegate patients’ population, avoiding biases related to selecting patients from a single institution. Third, the results of the systematic review and meta-analysis provide a complete synthesis of the literature on this topic and strengthen the results obtained from our sample. The limits of the study are its retrospective design and that, given the rarity of SMG involvement, it was not possible to stratify the patients according to the pathology characteristics.

Based on the results obtained in the present study, some conclusions may be drawn that can have direct implications in routine clinical practice. The incidence of SMG involvement in OCC seems to be possible but it occurs at extremely low rate, which justifies the possibility of gland sparing procedures, especially in early stage tumors with no involvement of the floor of the mouth or level I metastasis and in the treatment of the neck contralateral to the tumor. Consequently, a gland sparing protocol can be developed in which some patients have their glands preserved even when neck dissection is performed. If post-operative radiotherapy is chosen, coordination with the radiotherapist is fundamental to plan a sparing of the gland from high dose irradiation. Undoubtedly, future studies are needed in which a prospective comparison is made between spared and not spared groups to understand the oncological safety of gland preservation and its real impact on the quality of life.

## Supplementary Information

Below is the link to the electronic supplementary material.

Supplementary file1 (DOCX 18 KB)

Supplementary file2 (DOCX 14 KB)

## Data Availability

The data that support the findings of this study are available from the corresponding author upon reasonable request.
