# Supplementary material for: Submandibular gland involvement in oral cavity squamous cell carcinoma: a retrospective multicenter study
Source: Eur Arch Otorhinolaryngol. 2023 Jun 6;280(9):4205–14. doi: 10.1007/s00405-023-08007-8 (PMC10382344; doi:10.1007/s00405-023-08007-8)
Supplement: Supplementary file 1 — Supplementary file1 (DOCX 18 KB) [file 405_2023_8007_MOESM1_ESM.docx]

**Table S1**. Qualitative assessment of the included studies through the modified Newcastle–Ottawa Scale.

| **Reference/year** | **Question 1** | **Question 2** | **Question 3** | **Question 4** | **Question 5** | **Risk of bias** |
| --- | --- | --- | --- | --- | --- | --- |
| Agarwal, 2016 | Yes | Yes | Yes | No | Yes | Moderate |
| Ashfaq, 2014 | Yes | Yes | Yes | No | Yes | Moderate |
| Basaran, 2013 | Yes | Yes | Yes | Yes | Yes | Low |
| Byeon, 2009 | Yes | Yes | Yes | No | Yes | Moderate |
| Cakir-Cetin, 2018 | Yes | Yes | Yes | No | Yes | Moderate |
| Chen, 2009 | Yes | Yes | Yes | No | Yes | Moderate |
| Dhiwakar, 2010 | Yes | Yes | Yes | No | Yes | Moderate |
| Ebrahim, 2011 | Yes | Yes | Yes | Yes | Yes | Low |
| Fives, 2016 | Yes | Yes | Yes | Yes | Yes | Low |
| Jakhetiya, 2021 | Yes | Yes | Yes | Yes | Yes | Low |
| Javadi, 2021 | Yes | Yes | Yes | Yes | Yes | Low |
| Kruse, 2009 | Yes | Yes | Yes | Yes | Yes | Low |
| Malgonde, 2014 | Yes | Yes | Yes | Yes | Yes | Low |
| Malik, 2016 | Yes | Yes | Yes | Yes | Yes | Low |
| Mazarei, 2021 | Yes | Yes | Yes | Yes | Yes | Low |
| Naidu, 2011 | Yes | Yes | Yes | No | Yes | Moderate |
| Okoturo, 2012 | Yes | Yes | Yes | Yes | Yes | Low |
| Panda, 2015 | Yes | Yes | Yes | Yes | Yes | Low |
| Pasha, 2021 | Yes | Yes | Yes | Yes | Yes | Low |
| Razfar, 2009 | Yes | Yes | Yes | Yes | Yes | Low |
| Spiegel, 2004 | Yes | Yes | Yes | Yes | Yes | Low |
| Yang, 2020 | Yes | Yes | Yes | Yes | Yes | Low |
| Yang 2, 2020 | Yes | Yes | Yes | Yes | Yes | Low |
| Gu, 2020 | Yes | Yes | Yes | Yes | Yes | Low |
| Questions applied:  1. Did the patients represent the whole cases of the medical centre?  2. Was the diagnosis correctly made?  3. Were other important diagnoses excluded?  4. Were all important data cited in the report?  5. Was the outcome correctly ascertained? | | | | | | |
