# Supplementary material for: Submandibular gland involvement in oral cavity squamous cell carcinoma: a retrospective multicenter study
Source: Eur Arch Otorhinolaryngol. 2023 Jun 6;280(9):4205–14. doi: 10.1007/s00405-023-08007-8 (PMC10382344; doi:10.1007/s00405-023-08007-8)
Supplement: Supplementary file 2 — Supplementary file2 (DOCX 14 KB) [file 405_2023_8007_MOESM2_ESM.docx]

Search strategy for Pubmed (MEDLINE)

("submandibular gland"[MeSH] OR "submandibular gland invasion"[tw] OR "submandibular gland involvement"[tw]) AND ("Head and Neck Neoplasms"[MeSH] OR "Squamous Cell Carcinoma of Head and Neck"[MeSH] OR " carcinoma, squamous cell of head and neck "[MeSH] OR "Mouth Neoplasms"[MeSH] OR "oral cancer"[MeSH] OR "oropharynx cancer"[MeSH] OR "larynx cancer"[MeSH] OR "hypopharyngeal cancer"[MeSH]) AND ("analyses, survival"[MeSH] OR "prognosis”[MeSH] OR "recurrence”[MeSH] OR "prognostic factor”[tw] OR "outcome”[tw])

Search strategy for Scopus

TITLE-ABS-KEY ( "submandibular gland invasion" OR "submandibular salivary gland invasion" OR "submandibular gland metastasis" OR "submandibular gland involvement" ) AND TITLE-ABS-KEY ( "Head and Neck Neoplasms" OR "Squamous Cell Carcinoma of Head and Neck" OR " oral cavity carcinoma" OR "oropharynx carcinoma" OR "Mouth Neoplasms" OR "oral cancer" OR "oropharynx cancer" OR "larynx cancer" OR "hypopharyngeal cancer" OR "hypopharynx cancer" ) AND ( LIMIT-TO ( LANGUAGE , "English" ) )
